# Prevalence and Risk Factors of Dengue Virus Infection Among Febrile Patients in a Malaria Hyperendemic Area of Southern Mali

**DOI:** 10.21203/rs.3.rs-9888571/v1

**Published:** 2026-06-18

**Authors:** Sidy Bane, Bourama Keita, Mohamed Seyba Coulibaly, Salif Thiam, Hamady Sissoko, Badara Aliou Konaté, Moussa Fouré, Sambou Diaby, Bonheur Dounebaine, Moussa Keita, Abdoul Fatao Diabate, Kyle Rosenke, Nafomon Sogoba, Heinz Feldmann, Seydou Doumbia

**Affiliations:** International Center of Excellence in Research (ICER) in Mali; International Center of Excellence in Research (ICER) in Mali; International Center of Excellence in Research (ICER) in Mali; International Center of Excellence in Research (ICER) in Mali; International Center of Excellence in Research (ICER) in Mali; International Center of Excellence in Research (ICER) in Mali; International Center of Excellence in Research (ICER) in Mali; International Center of Excellence in Research (ICER) in Mali; Public Health Institute and Research, Africa CDC, Africa Union;; International Center of Excellence in Research (ICER) in Mali; International Center of Excellence in Research (ICER) in Mali; Laboratory of Virology, Division of Intramural Research, National Institute of Allergy and Infectious Diseases, National Institutes of Health; International Center of Excellence in Research (ICER) in Mali; Laboratory of Virology, Division of Intramural Research, National Institute of Allergy and Infectious Diseases, National Institutes of Health; University Clinical Research Center (UCRC)

**Keywords:** Febrile patients, Dengue infection, Diagnosis, Prevalence, Risk factors, Mali

## Abstract

**Background:**

Dengue virus is a major cause of febrile illness and is often misdiagnosed as malaria in regions lacking appropriate diagnostics. This study assessed the prevalence and risk factors for dengue infection among febrile patients attending community health centers in the malaria hyperendemic Sikasso region of southern Mali.

**Methods:**

From June (beginning of the rainy season) to December (post rainy season) 2023, patients presenting with fever at four community health centers (Bamba, Fakola, Filamana, and Guélélenkoro) were tested for dengue infection using rapid diagnostic tests. A multivariate logistic regression model was applied to identify risk factors associated with symptomatic infection.

**Results:**

Of 251 enrolled patients, 63.0% were male, and the median age was 15 years (± 13.7). The prevalence of dengue infection varied between 9.7% to 47.8% with the highest observed in December after the rainy season. Headache, muscle pain, and abdominal pain were the most common clinical symptoms associated with dengue fever. Children 5–15 years had significantly higher risk of infection compared to those under 5 years of age (OR = 7.4; 95% CI [1.44; 38.26]). The risk of infection was highest in November (OR = 4.29; 95% CI [1.12; 16.48]) and December (OR = 10.30; 95% CI [2.56; 41.36]).

**Conclusion:**

Dengue infections contribute substantially to febrile illness in southern Mali, particularly among school-aged children, mainly during the late rainy season and early dry season. The finding highlights the need for integration of dengue testing into routine diagnostic protocols for febrile patients in malaria-endemic rural settings to reduce misdiagnosis and improve patient outcomes.

## Background

Dengue virus (DENV), transmitted by *Aedes* mosquitoes is a major public health problem in tropical and subtropical regions. Dengue infections cause a spectrum of illness ranging from asymptomatic to milder Dengue Fever (DF) to severe and fatal Dengue Hemorrhagic Fever (DHF) or Dengue Shock Syndrome (DSS) (1, 2). There are four distinct serotypes (DENV-1, DENV-2, DENV-3, and DENV-4) all of which may cause disease. Severe illness often follows a previous dengue infection with a different serotype and the presence of heterologous dengue-specific antibodies (3). Current dengue vaccines do not provide long-lasting immune protection against all four dengue serotypes, and there are no antiviral treatments (4). Prevention relies primarily on vector control measures to reduce mosquito bites.

According to the World Health Organization (WHO), 171,991 suspected cases, including 70,223 confirmed/probable cases and 753 deaths, were recorded in 15 African countries in 2023, which is a ninefold increase compared to 2019 (5). All 4 dengue serotypes are present in Africa, although DENV-2 has caused the most recent epidemics on the continent. *Aedes aegypti*, the vector that transmits DENV, is reported in 47 of 55 African countries (6).

In Mali, seroprevalence studies have shown that DENV circulates in several regions, with sporadic outbreaks of DF (7–9). Between September 2023 and December 2024, Mali experienced its largest documented dengue outbreak, with cases reported from all 11 administrative regions, resulting in 16,355 suspected cases, including 1,763 confirmed cases and 74 deaths (10). Serotypes DENV-1 and DENV-3 have been associated with these epidemics and DENV-3 represented about 80% of human cases during the 2023 Outbreak (11). Despite documented seroprevalence and clinical DF cases in Mali, few studies have assessed the burden of dengue among febrile patients, particularly in rural settings where there is limited diagnostic capacity to differentiate DF from other infectious diseases, especially malaria. This study aimed to determine the prevalence of dengue infection among febrile patients seeking care at rural community health centers in southern Mali, where malaria is hyperendemic.

## Material and Methods

### Study setting

**Figure 1:**
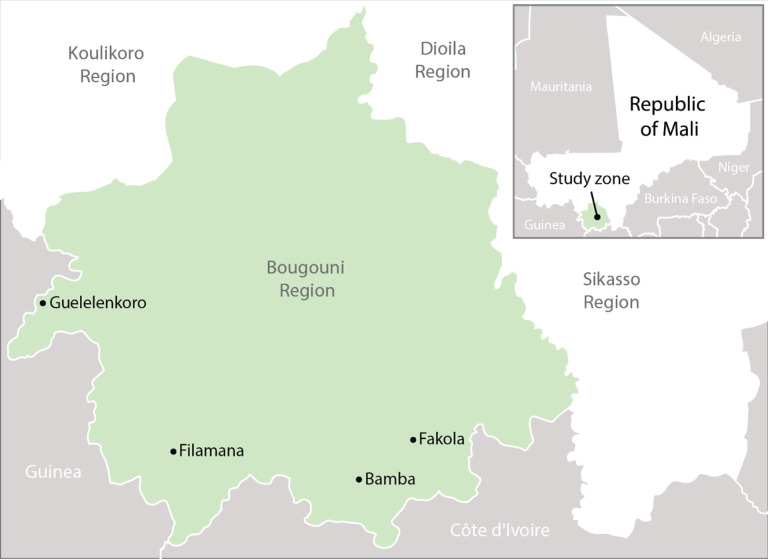
Map showing the four study sites in Southern Mali.

The study was conducted in four community health centers (CSComs) located in the district of Bougouni, region of Sikasso, in southern Mali (Fakola, Bamba, Guélélenkoro, and Filamana). These sites were selected based on previous serological studies that provided evidence of high exposure to DENV (9). The population size covered by these community health centers was 13,082 inhabitants for Bamba (140 km from the referral district hospital of Bougouni), 16,681 inhabitants for Fakola (75 kilometers from the referral district hospital of Kolondièba), 12,028 inhabitants for Filamana (95 kilometers from the referral district hospital of Yanfolila), and 6,462 inhabitants for Guélélenkoro (40 kilometers from the referral district hospital of Yanfolila). Geographically, Bamba, Fakola and Filamana are located along the border with Cote d’Ivoire, whereas Guélélenkoro is located along the border with Guinea (Fig. 1). Southern Mali is characterized by a long rainy season (April through October) with high annual rainfall ranging from 1,200 to 1,600 mm. The dry season is characterized by moderate temperatures (20 to 30°C) from November to February, and hot temperatures in March-April (30–35°C). The average annual temperature is approximately 28°C. The local vegetation primarily consists of wooded savannah, gallery forests along rivers, and areas of dense forest.

#### Study participants

We conducted a cross-sectional study among febrile patients seeking care at the four CSComs between June and December 2023. In Mali, CSComs are primary healthcare facilities and the first level of the country’s health system, preceding referral district hospital or Reference Health Centers. CSComs provide basic preventative and curative medical services to local populations through a general medical doctor or nurses and are managed by Community Health Associations (ASACOs). A CSComs typically covers a population of around 10,000–20,000 within a radius of 15–20 kilometers. We enrolled all patients seeking care at the CSComs with fever at admission (body temperature ≥ 38°C) and at least two of the following symptoms: headache, myalgias, arthralgias, skin rash, facial flushing, skin erythema, anorexia, nausea, vomiting, or bleeding. For each patient, sociodemographic information such as age, sex, address, and clinical history was recorded.

#### Sampling and rapid diagnostic testing

Blood samples were collected via finger prick into EDTA tubes and tested for dengue and malaria using rapid diagnostic tests (RDT). For the diagnosis of dengue, we used the “Dengue Test DAY 1” rapid diagnostic test (*Mitra & Co. Pvt Ltd, India*) that detects the presence of *NS1* antigen, IgM and IgG antibodies in a plasma sample without distinguishing the specific serotype. The test detects dengue infection within the first few days of fever onset, helping to differentiate primary and secondary infections. According to the manufacturer, the sensitivity and specificity for DENV *NS1* antigen are 96.0% and 98.0%, respectively. The sensitivity and specificity of the DENV IgG/IgM antibody test are 95% and 97%, respectively (12). An acute dengue infection was defined as fever and a positive IgM antibody and/or NS1 antigen test result. For malaria diagnosis, we used the rapid diagnostic Bioline^™^ Malaria Ag *P.f*/Pan test (Abbott Diagnostics Korea Inc). Depending on the antigen, the performance is as follows: *P.f* (HRP2) with a sensitivity of 99.7 and a specificity of 99.5%; and Pan (pLDH) with a sensitivity 95.5% and a specificity 99.5% (13). This test is a qualitative and differential test that detects the histidine-rich protein 2 (HRP2) antigen of *Plasmodium falciparum* and the common *Plasmodium lactate dehydrogenase* (pLDH) of other malaria species. Malaria was defined as fever and a positive RDT. RDTs were performed following protocols provided by the manufacturers.

### Data analysis

Data was collected through case report forms, entered into Microsoft Excel, and analyzed with Stata 15.1. Association between dengue infection (IgM and/or NS1 positive) and potential risk factors were first assessed using bivariate binary logistic regression. The risk factors tested included demographic variables (age, sex, and place of residence), clinical symptoms (headache, myalgia, arthralgia, abdominal pain, nausea/vomiting, rash, and bleeding), seasonality (month of presentation, June to December), and malaria co-infection (presence or absence of malaria by RDT). These variables were selected based on biological plausibility and prior evidence linking demographic, clinical, and seasonal factors to dengue transmission and disease severity. Variable with p ≤ 25% in bivariate analysis were entered into a multivariate logistic regression model to identify independent predictors of dengue infection. Pearson’s Chi-squared test or Fisher’s Exact test were applied where appropriate. The level of association was determined by calculating the adjusted odds ratios (OR) and the 95% confidence interval. The significance level was set at p < 0.05.

## Results

### Socio–demographic characteristics of febrile study participants

Between June 1st and December 30, 2023, a total of 12,695 people sought care at the four study sites. Of these 251 febrile patients who met the inclusion criteria were enrolled. Among these, 63.95% were male, the predominant age group was 5 to 15 years old (40.24%), and the median age was 15 years (± 13.7). More than half of the study participants (58.57%) were from Bamba, 11.95% from Filamana, 20.72% from Guélélenkoro, and 8.76% from Fakola. Regarding sociodemographic characteristics, 42.23% were married, 36.25% were farmers, 15.54% were housewives, and 48.21% were children. These characteristics are summarized in Table 1. Due to low number of participants per study site, data from all CSComs were combined in the subsequent analyses.

#### Dengue status among febrile study participants

Of the 251 febrile patients enrolled, 22.7% (57 cases) tested positive for dengue by RDT (NS1and/or IgM). Among these, 71% (41 cases) were also malaria RDT positive, highlighting the high prevalence of co- infection with *plasmodium ssp*. Only 6.3% (16 cases) were diagnosed with dengue alone, while nearly 70% (175 cases) were diagnosed with malaria. A small proportion (7.6%, 19 cases) tested negative for both malaria and dengue RDTs, confirming that malaria as the dominant cause of fever in this area. Bivariate analysis revealed a significant association between dengue positivity and age group, with children aged 5 to15-years-old more likely to be infected (p = 0.03). No significant associations were found with other socio-demographic variables. These findings are presented in Table 2.

## Discussion

Dengue infections are increasingly recognized as a cause of febrile illness and are becoming highly endemic across sub-Saharan Africa (14). Serological studies have suggested dengue circulation in many regions of Mali (7–9), and between September 2023 and December 2024, the country experienced its largest and longest dengue outbreak to date (11, 15). Dengue infections continue to be reported regularly and became endemic in Mali. While most cases are reported in urban settings, underreporting in rural areas is likely due to limited diagnostic capacity and frequent misclassification as malaria. This study provides the first evidence of dengue infections as a significant contributor to febrile illness in rural settings of southern Mali, where high dengue IgG seroprevalence has previously been reported (9).

IgM antibodies typically rise after primary dengue infection, while IgG increase during secondary infections and indicates prior exposure (16). The RDT used in this study detects dengue NS1 antigen, IgM and IgG allowing to distinguish between acute past infections (17). In this study, more than 75% of dengue RDT positive cases showed IgM antibodies and 14% displayed both IgM and IgG. Unlike earlier population-based seroprevalence surveys that showed high dengue IgG seroprevalence (9), our study focused on symptomatic patients who may have developed an acute primary or secondary dengue infection. Infection with one dengue serotype confers long-lasting immunity against that serotype but does not protect against subsequent infections with different serotypes (18). Although circulating serotypes were not determined, DENV-1 and DENV-3 have been widely reported in Mali and in the subregion (11, 15). Secondary infections increase the risk of severe DHF and DSS (19, 20), both of which have high case fatality rates, highlighting the need for dengue genomic surveillance in rural areas to better define serotype distribution and assess the risk for severe disease.

A monthly variation was observed with infection rising after the end of the rainy season, accounting for nearly half of the febrile cases (47.8%) in December. During the recent epidemic in Mali, particularly in Bamako, dengue cases peaked in October to November 2023, and this trend was replicated in 2024, coinciding with the end of the rainy season (15). A delay between heavy rainfall and the peak of dengue transmission was also reported in the neighboring country of Burkina Faso (21, 22). In West Africa, DENV transmission peaks between September and December (23), Several factors are associated with the increasing risk of the spread of the dengue epidemic, including heavy rainfall, high humidity, and rising temperatures (24).. The gallery forests and woodland savannah of southern Mali provide an ideal habitat for *Aedes* mosquitoes supporting the potential for year-round dengue transmission with surge after the end of the rainy season (November through December).

Clinically, the most common symptoms among dengue patients were headache (86.85%), abdominal pain (86.06%), and muscle ache (89.64%), overlap substantially with malaria symptom, complicating differential diagnosis. No cases of DHF or DSS were observed, though the small sample size limits conclusion about severe diseases.

Co-infection with malaria was frequent, with over 70% of the dengue-positive patients also testing positive for *Plasmodium spp*. Previous meta-analysis reported lower co-infection rates across Africa 4.2%, with West Africa showing the lowest rate (1.6%) (25). Our higher prevalence of coinfection (16.3%) likely reflects hyperendemic malaria transmission in the study area, where the prevalence of parasitemia ranges from 40–50% during the dry season to 70–85% during the rainy season (9, 26, 27). Rising rate of *P. falciparum*-DENV coinfection have been documented continent-wide, increasing from 0.9% (2008–2013) to 3 to 5.5% (2018–2021) (25). With dengue infections increasing in Mali, further studies are needed to estimate the burden of coinfection with DENV -*Plasmodium spp*. in malaria-hyperendemic areas.

The 5- to 15-year-old individuals had a significantly higher risk of dengue infection compared to the younger age group (< 5-year-old). This finding is consistent with the seroprevalence survey conducted in the study areas, which showed more than 75–80% dengue IgG seroprevalence in adults above 30 years old compared with children and adolescents (9). Data from the national surveillance found that the 20–24 year age group was the most affected in the 2023–2024 epidemic (15). Cases from this surveillance data are mostly from urban areas with different risks of exposure compared to rural settings, where the population is exposed to dengue at an early age. Further investigations are needed to determine the age-related risk factors for DF.

Limitations of this study include potential selection bias, as more severe febrile cases are more likely to seek care at the CSComs, unintentionally underestimating the true burden of dengue in the communities. Limited blood amount from fingerstick prevented RT-PCR confirmation and serotyping. Finally, the study enrollment did not run throughout an entire calendar year, limited conclusion about year-round transmission. Despite these limitations, our findings demonstrate that dengue is a significant public health burden in southern Mali. Longer study incorporating molecular diagnostics and genomic surveillance are needed to better characterize transmission dynamics, serotype distribution, and the burden of co-infection with malaria.

## Conclusion

This study demonstrates that dengue is a significant contributor to febrile illness in rural southern Mali, particularly among school-aged children, and peaks during the late rainy and early dry seasons. The high rate of co-infection with malaria underscores the diagnostic challenges in hyperendemic settings and highlights the urgent need to integrate dengue testing into routine fever case management. Strengthening surveillance systems, expanding diagnostic capacity, and incorporating genomic monitoring of circulating serotypes will be essential to improve patient outcomes and guide public health interventions in Mali and across West Africa.

## Figures and Tables

**Figure 2:**
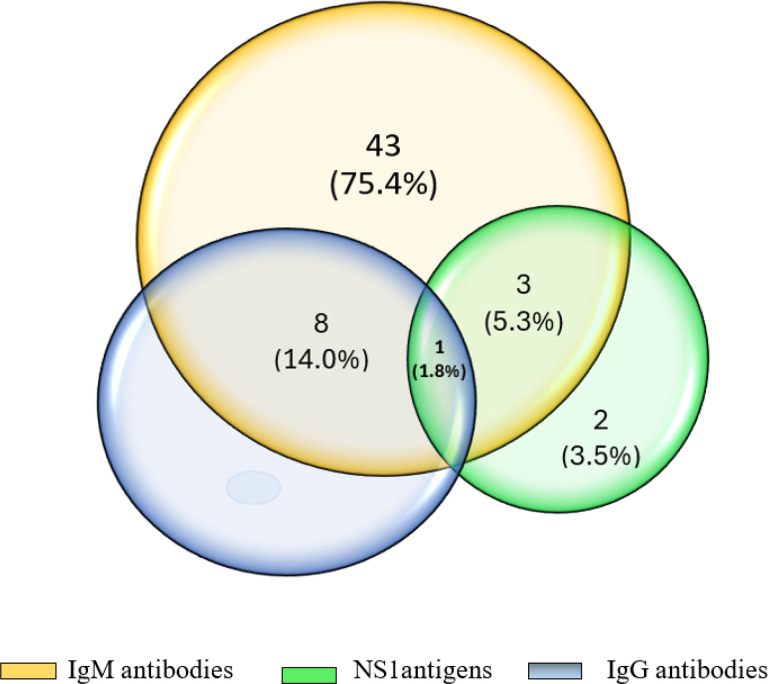
Venn diagram showing positivity for either NS1 antigen, IgM antibodies, and/or IgG antibodies.

**Figure 3:**
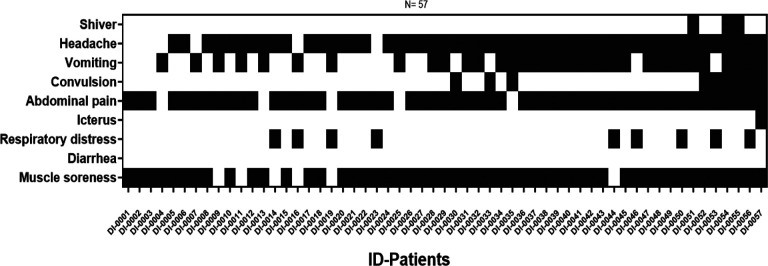
Frequency of clinical symptoms among dengue-positive participants

**Figure 4:**
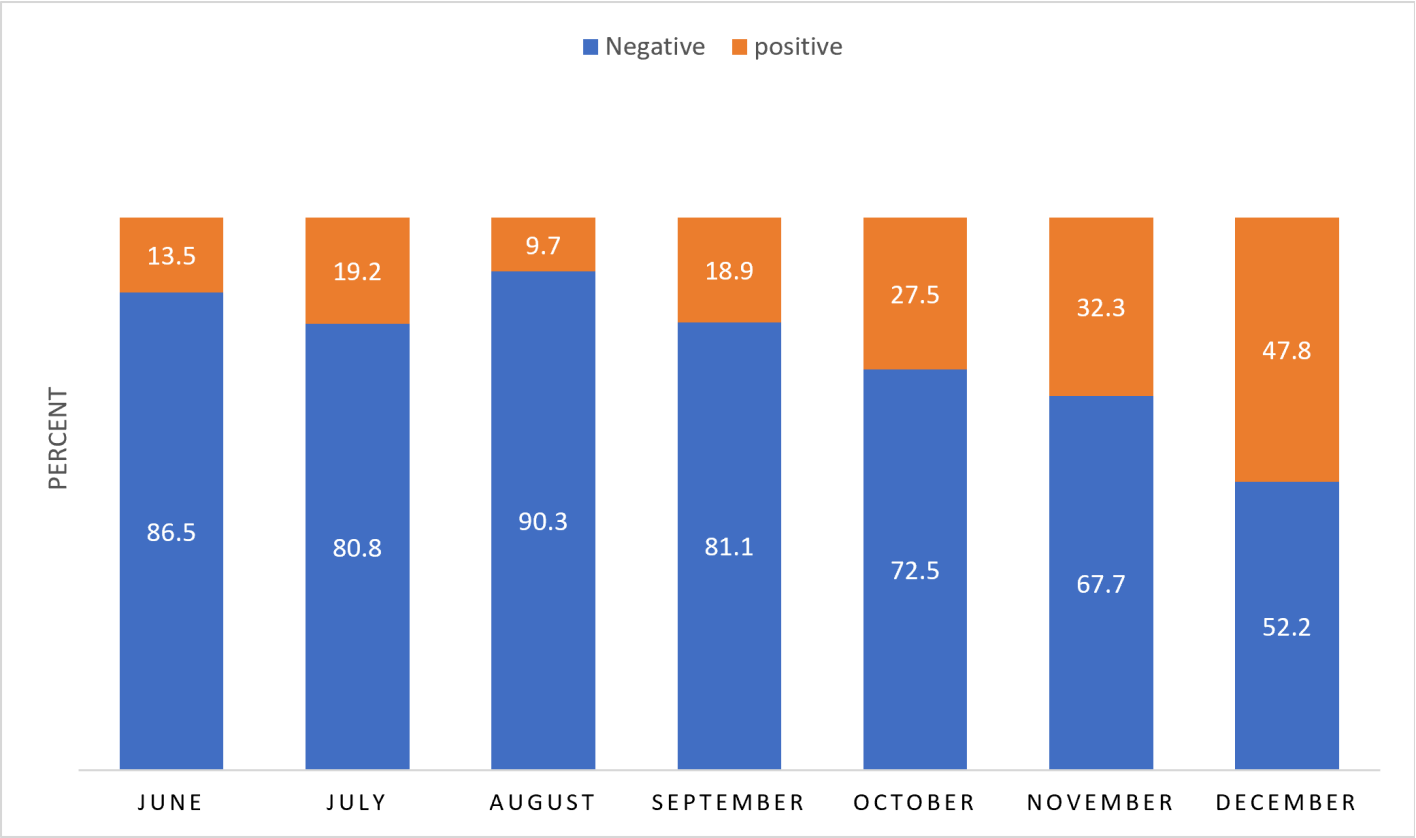
Distribution of dengue infections by month

**Table 1:** Socio-demographic characteristics of febrile study participants from four rural communities in southern Mali

Socio-demographic characteristics	Number N = 251	Percentage
**Sex**		
Male	158	62.95
Female	93	37.05
**Age group**		
Under 5 years	26	10.36
5 to 15 years	101	40.24
16 to 25 years	53	21.11
26 to 35 years	39	15.54
36 years and more	32	12.75
**Study site**		
Bamba	147	58.57
Filamana	30	11.95
Guélélenkoro	52	20.72
Fakola	22	8.76
**Matrimonial status**		
Married	106	42.23
Single	24	9.56
Child	121	48.21
**Profession**		
Farmer	91	36.25
House-wife	39	15.54
Child	121	48.21
Total	251	100

**Table 2:** Bivariate analysis of socio-demographic characteristics and dengue status.

Socio-demographic characteristics	Dengue positive n (%)	Dengue negative n (%)	p-value
**Sex**			
Male	35 (22.15)	123 (77.85)	0.78
Female	22 (23.66)	71 (76.34)	
**Age group**			
Under 5 years	2 (7.70)	24 (92.31)	-
5 to 15 years	28 (27.72)	73 (72.28)	0.03
16 to 25 years	13 (24.53)	40 (75.47)	0.12
26 to 35 years	7 (17.95)	32 (82.05)	0.43
36 years and more	7 (21.85)	25 (78.13)	0.26
**Matrimonial status**			
Married	21 (19.81)	85(80.19)	-
Single	6 (25.00)	18(75.00)	0.75
Child	30 (24.79)	91(75.21)	0.36
**Profession**			
Farmer	21 (23.08)	70(76.92)	-
House-wife	6 (15.38)	33 (84.62)	0.32
Child	30 (24.80)	91 (75.21)	0.77
Total	57(22.70)	194 (77.29)	

## Data Availability

The data sets used and/or analyzed during the current study are available from the corresponding author upon request.
